# Morphological characteristics and phylogenetic analyses revealed four new species of Agaricales from China

**DOI:** 10.3389/fmicb.2023.1118525

**Published:** 2023-02-03

**Authors:** Jize Xu, Yi Jiang, Tiantian Wang, Di Zhang, Xiaobin Li, Md. Iqbal Hosen

**Affiliations:** ^1^Agricultural College, Jilin Agriculture Science and Technology University, Changchun, Jilin, China; ^2^College of Plant Science, Jilin University, Changchun, Jilin, China; ^3^Agricultural College, Yanbian University, Yanji, Jilin, China; ^4^College of Biodiversity Conservation and Utilization, Southwest Forestry University, Kunming, China

**Keywords:** four new species, fungi, morphological studies, mushrooms, phylogenetic analysis, taxonomy

## Abstract

Four new species of Agaricales from China viz. *Hohenbuehelia tomentosa, Rhodophana qinghaiensis, Rhodophana aershanensis*, and *Spodocybe tomentosum* are described based on their unique morphological features and molecular evidence. *Hohenbuehelia tomentosa* is mainly characterized by its dark brown pileus with finely dense pure white tomentum, dirty white, decurrent lamellae, eccentric stipe, smooth spores, and fusiform metuloid cystidia. The characteristics of *Rhodophana qinghaiensis* are glabrous, smooth, reddish-brown pileus, gray-orange lamellae, and initially light orange becoming reddish brown stipe. The unique morphological characteristics of *Rhodophana aershanensis* are reddish brown pileus with age, brown-orange toward the margin, light orange lamellae and stipe dark brown at first, and reddish-brown with age. *Spodocybe tomentosum* is characterized by subclitocyboid and small basidiomes, finely dense pure white tomentum on the pileus surface, and broadly ellipsoid to ellipsoid and smaller basidiospores. Phylogenetic analysis showed that *Hohenbuehelia tomentosa, Rhodophana qinghaiensis, Rhodophana aershanensis*, and *Spodocybe tomentosum* formed an independent lineage. Full descriptions, illustrations, and phylogenetic trees of the four new species are provided in this study.

## 1. Introduction

In recent years, many new species have been reported in China. For example, Liu et al. (2010) published three new records in China [*Hohenbuehelia angustata* (Berk.), Singer., *H. nigra* (Schwein.) Singer., and *H. grisea* (Peck) Singer.]. Similarly, Yang and Fan (2020) published a new species (*Rhodophana guandishanensis* L. Fan and C. Yang.) and He and Yang (2021) published two new species [*Spodocybe bispora* Z. M. He and Zhu L. Yang., *S. rugosiceps* Z. M. He and Zhu L. Yang.], all in China. This article introduces four new species from *H. tomentosa, R. qinghaiensis, R. aershanensis*, and *S. tomentosum*.

The Agaricales is the largest and most diverse order of mushroom-forming Basidiomycota, including *Crepidotus* (Fr.) Staude. (Kumar et al., 2018), *Clavaria* P. Micheli. (Yan et al., 2020), *Hohenbuehelia* Schulzer. (Kirk et al., 2001), *Rhodophana* Kühner. (Daniëls et al., 2017), *Spodocybe* Z. M. He & Zhu L. Yang. (He and Yang, 2021). *Hohenbuehelia* Schulzer. is nematophagous, has 135 valid records in Index Fungorum, and is distributed across Canada, Austria, the US, and China (Liu et al., 2010; Consiglio et al., 2018). *Hohenbuehelia* was established and first described by Schulzer von Müggenburg et al. (1866) and belongs to Pleurotaceae Kühner., Agaricales Underw. (Kirk et al., 2001). It is mainly characterized by spathulate, reniform, or flabelliform pileus, usually with a gelatinous layer under the surface of the pileus, with decurrent lamellae, diminished or no stipe, ellipsoid basidiospores, and thick-walled cystidia. In earlier studies, the genus *Hohenbuehelia* was placed under the genus *Pleurotus* (Fr.) P. Kumm. due to the similar pileus shape (conchiform) (Pilát and Veselý, 1935). However, the results of phylogenetic analyses based on 25S rDNA indicated that *Hohenbuehelia* differs from *Pleurotus* and exists as a monophyletic genus in Pleurotaceae (Thorn et al., 2000). Mentrida (2016) also proposed that *Hohenbuehelia* and *Pleurotus* could be distinguished from each other by the presence of a gelatinous layer between the context and the cuticle (present in the *Hohenbuehelia* species, but not in the *Pleurotus*). The asexual stage was separately described in the anamorph genus *Nematoctonus* Drechsler. (Drechsler, 1941; Thorn and Barron, 1986). Based on the phylogenetic analyses of ITS + nrLSU sequences, *Nematoctonus* and *Hohenbuehelia* are currently considered different (Koziak et al., 2007). According to the naming rules for fungi adopted in Melbourne in 2011 (Taylor, 2011), all members of the genus should therefore be referred to as *Hohenbuehelia* (Thorn, 2013). However, as more and more species are described in *Hohenbuehelia*, different researchers have inconsistent views on the infrageneric classification.

*Rhodophana* is a genus that was defined from Entolomataceae Kotl & Pouzar in 2014 (Kluting et al., 2014) and distributed in Canada, Argentina, Australia, the US, and China (Yang and Fan, 2020). The genus was first established by Kühner with *R. nitellina* (Fr.) T. J. Baroni & Bergemann. as the type species (Kühner, 1947). At the time, it was not effectively published until Kühner redescribed it in 1971, during which it was described as a *Rhodocybe* subgenus (Kühner and Lamoure, 1971). In 2014, based on phylogenetic analysis, Kühner found that it belonged to an independent branch, so it was repositioned as a genus. According to the current research, it was predicted that there will be many new combinations of this genus (Buyck et al., 2021). The morphological characteristics of this genus are mainly the collybioid basidiocarps, adnexed to adnate lamellae, and basidiospores with undulate-pustulate ornamentation, which is observed in the direction of the polar view of the angle, and more or less clamp connection. Since its establishment, there are 19 records in Index Fungorum, of which 15 are valid records, distributed in Europe [Spain and France] (Vizzini et al., 2012; Buyck et al., 2021), Asia [India and China] (Raj et al., 2016; Yang and Fan, 2020), and Canary Island of Africa (Vizzini et al., 2011).

Currently, *Spodocybe* is distributed in China and Europe (He and Yang, 2021; Vizzini et al., 2021). *Spodocybe* has the characteristics of saprophytic life, usually gregarious or caespitose on the ground of coniferous or coniferous and broad-leaved mixed forest and distributed in temperate and subtropical zones from June to November. Lodge et al. (2014) revised the agaric family Hygrophoraceae Lotsy. while retaining the Cuphophylloid rank in the family Hygrophoraceae as the basis for the composition of *Cuphophyllus* (Donk) Bon., *Ampulloclitocybe* Redhead, Lutzoni, Moncalvo & Vilgalys. and *Cantharocybe* H. E. Bigelow & A. H. Sm.e. (Matheny et al., 2006; Binder et al., 2010; Lodge et al., 2014). However, the taxonomic problems of these three genera remained unresolved due to weak phylogenetic support. In 2021, *Spodocybe* was established as a new genus by He and Yang (2021) and placed in the new subfamily, Cuphophylloideae Z. M. He & Zhu L. Yang., which consists of *Spodocybe, Ampulloclitocybe, Cantharocybe*, and *Cuphophyllus. Spodocybe* is mainly characterized by clitocyboid basidiomes, decurrent lamellae, inamyloid basidiospores, lamellar trama subregular, and the presence of clamps connections. According to the Index Fungorum, *Spodocybe* contains six species, namely *S. bispora, S. collina* (Velen.) Vizzini, P. Alvarado & Dima, *S. fontqueri* (R. Heim) Vizzini, P. Alvarado & Dima., *S. herbarum* (Romagn.) Vizzini, P. Alvarado & Dima., *S. rugosiceps*, and *S. trulliformis* (Fr.) Vizzini, P. Alvarado & Dima.

The present study aims to describe four new species viz. *H. tomentosa, R. qinghaiensis, R. aershanensis*, and *S. tomentosum*.

## 2. Materials and methods

### 2.1. Specimen collection

All the specimens were collected from China. The collected samples were dried overnight using an electric drying oven at 45°C and deposited in the Herbarium Mycology of Jilin Agricultural Science and Technology University (HMJU).

### 2.2. Morphological analysis of specimens

The macromorphological descriptions were based on field notes and photographs captured in the field. Images of the samples were taken in natural light using a Canon camera and tripod. The color card described by Kornerup and Wanscher (1978) was used. The micromorphology of the specimens was studied under an Olympus BX 53 (Tokyo, Japan) light microscope at 40, 100, 400, 600, and 1,000 × magnifications. All measurements were observed under a 1,000 × oil immersion. Sections of the dried specimens were mounted in 3% KOH, Melzer's reagent, Congo red, and Cotton blue for observations. Cotton blue and iron acetocarmine solutions were utilized to highlight the siderophilous granulation in the basidia following the study of Baroni (1981).

Basidiospore measurements were made by photographing all spores from time to time (taken from dry specimens) and measured *via* Cellsens Standard. The spore dimensions excluded the hilar appendix and the ornamentation and are given as (minimum–) average minus standard deviation—average plus standard deviation (–maximum) of length × (minimum–) average minus standard deviation—average plus standard deviation (–maximum) of width. Factor Q is the ratio of spore length to width, and Qm is the average of factor Q. When calculating the average, the maximum and minimum values are discarded. The scanning electron microscope (SEM) procedure was the one followed by Xu et al. (2019).

### 2.3. DNA extraction, PCR amplification, and DNA sequencing

Genomic DNA was extracted from the dried samples following the procedure described by Zhao et al. (2011). For the polymerase chain reaction (PCR) amplification, primer pair ITS1 and ITS4 (White et al., 1990) were used for the internal transcribed spacer (ITS). Primer LR0R was paired with LR5 and LR7 (Vilgalys and Hester, 1990) to obtain sequences for the nuclear ribosomal large subunit (nrLSU). Primer pairs 5F and 7.1R (Matheny, 2005) and RhoF1 and RhoR1 (Kluting et al., 2014) were used for the DNA-directed RNA polymerase II second largest subunit 2 (*rpb2*). Primers ATP6-1 and ATP6-2 (Kretzer and Bruns, 1999) were used for the ATP synthase subunit 6 (*atp6*) and primers 526F and 1567R (Matheny et al., 2007), and 595F (Wendland and Kothe, 1997) and Efgr (Rehner and Buckley, 2005) were used for the translation elongation factor 1-α (*tef1-*α). The reaction program is shown in Table 1. The PCR products were examined on a 1% agarose gel detected by a JY 600 electrophoresis apparatus (Beijing JUNYI Electrophoresis Co., Ltd., Beijing, China) and then sent to BGI Co., Ltd. (Beijing, China) for sequencing.

**Table 1:** List of PCR used in this study.

**Species name**	**Sequence name**	**Primer pair**	**PCR amplification condition**	
*Hohenbuehelia tomentosa*	ITS	ITS1/ITS4	94°C 4 min 94°C 1min 56°C 1 min 72°C 1 min 72°C 5 min	40 cycles
	nrLSU	LROR/LR7	94°C 4 min 94°C 90 s 56°C 45 s 72°C 90 s 72°C 5 min	40 cycles
*Rhodophana qinghaiensis*	*rpb2*	RhoF1/RhoR1	95°C 3 min 94°C 30 s 58°C 60 s 72°C 90 s 72°C 8 min	35 cycles
	*tef1-α*	EF1-526F/EF1-1567R	94°C 3 min 94°C 30 s 52°C 30 s 72°C 30 s 72°C 3 min	33 cycles
*Rhodophana aershanensis*	*atp6*	ATP6-3/6r	95°C 5 min 95°C 30 s 42°C 2 min 72°C 1 min 72°C 10 min	33 cycles
	nrLSU	LROR/LR7	94°C 4 min 94°C 30 s 46°C 45 s 72°C 40 s 72°C 4 min	30 cycles
	*tef1-α*	EF1-595F/EF1-Efgr	94°C 3 min 94°C 30 s 54°C 30 s 72°C 1 min 72°C 10 min	32 cycles
*Spodocybe tomentosum*	ITS	ITS1/ITS4	94°C 5 min 94°C 30 s 52°C 30 s 72°C 30 s 72°C 5 min	33 cycles
	nrLSU	LROR/LR5	94°C 5 min 94°C 30 s 52°C 30 s 72°C 50 s 72°C 10 min	35 cycles
	*rpb2*	RPB2-5F/RPB2-7.1R	94°C 5 min 94°C 40 s 52.5°C 45 s 72°C 40 s 72°C 10 min	32 cycles
	*atp6*	ATP6-1/2	94°C 5 min 94°C 40 s 52.5°C 45 s 72°C 40 s 72°C 10 min	32 cycles

### 2.4. Phylogenetic analyses

**Figure 1:**
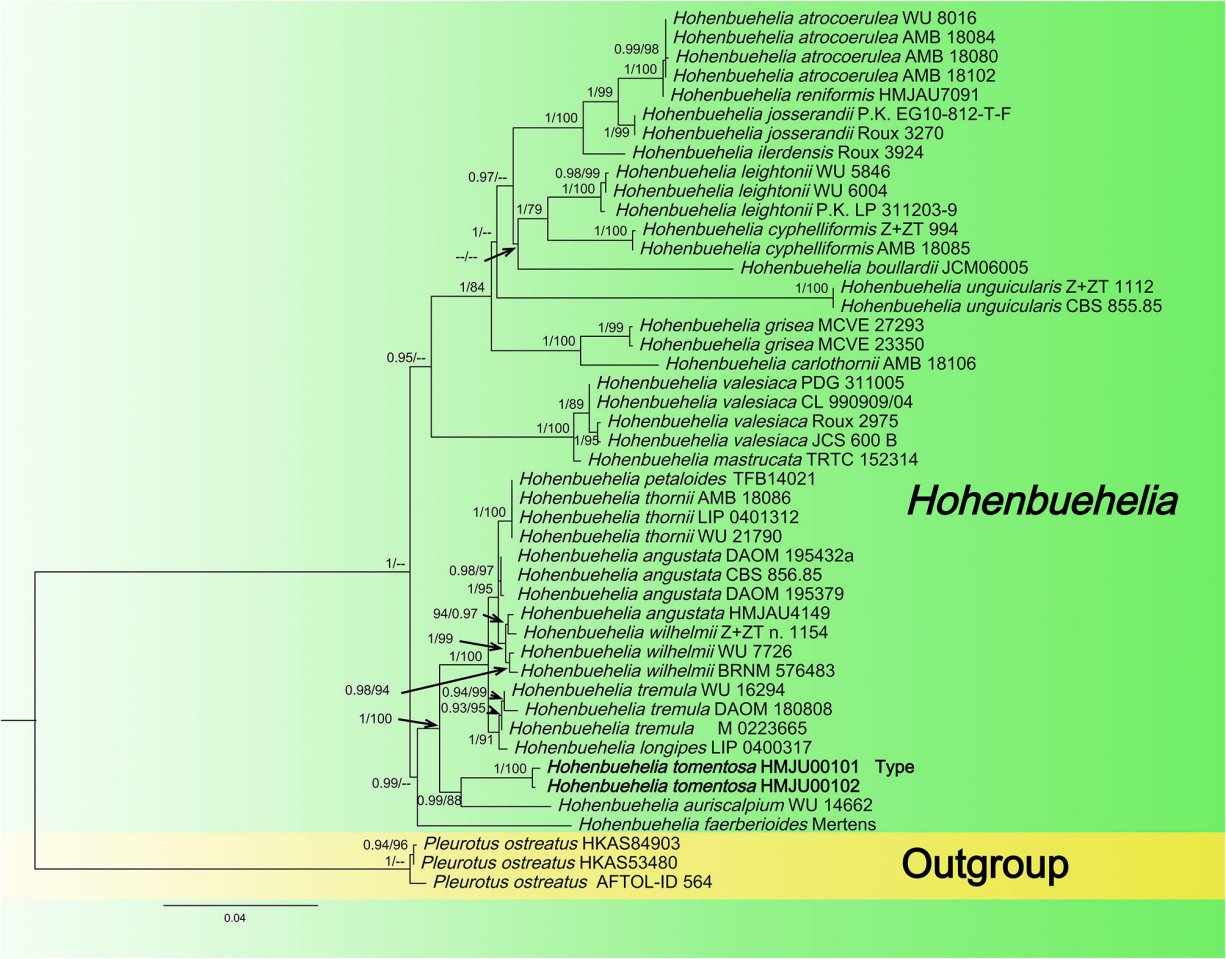
Maximum likelihood tree based on analyses of the ITS and nrLSU sequence data.

**Figure 2:**
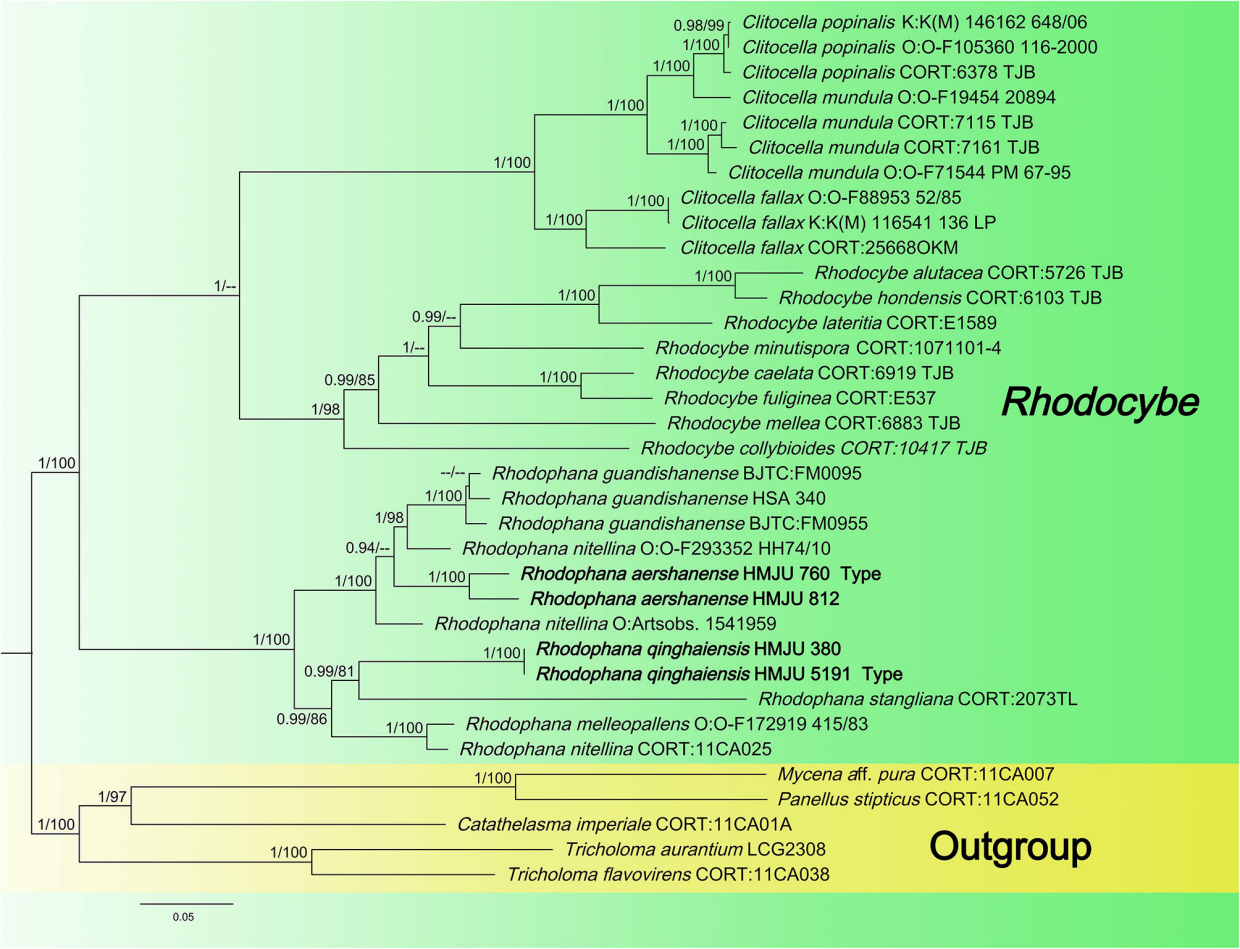
Maximum likelihood tree based on analyses of the nrLSU, *rpb2*, and *tef1-*α sequence data.

**Figure 3:**
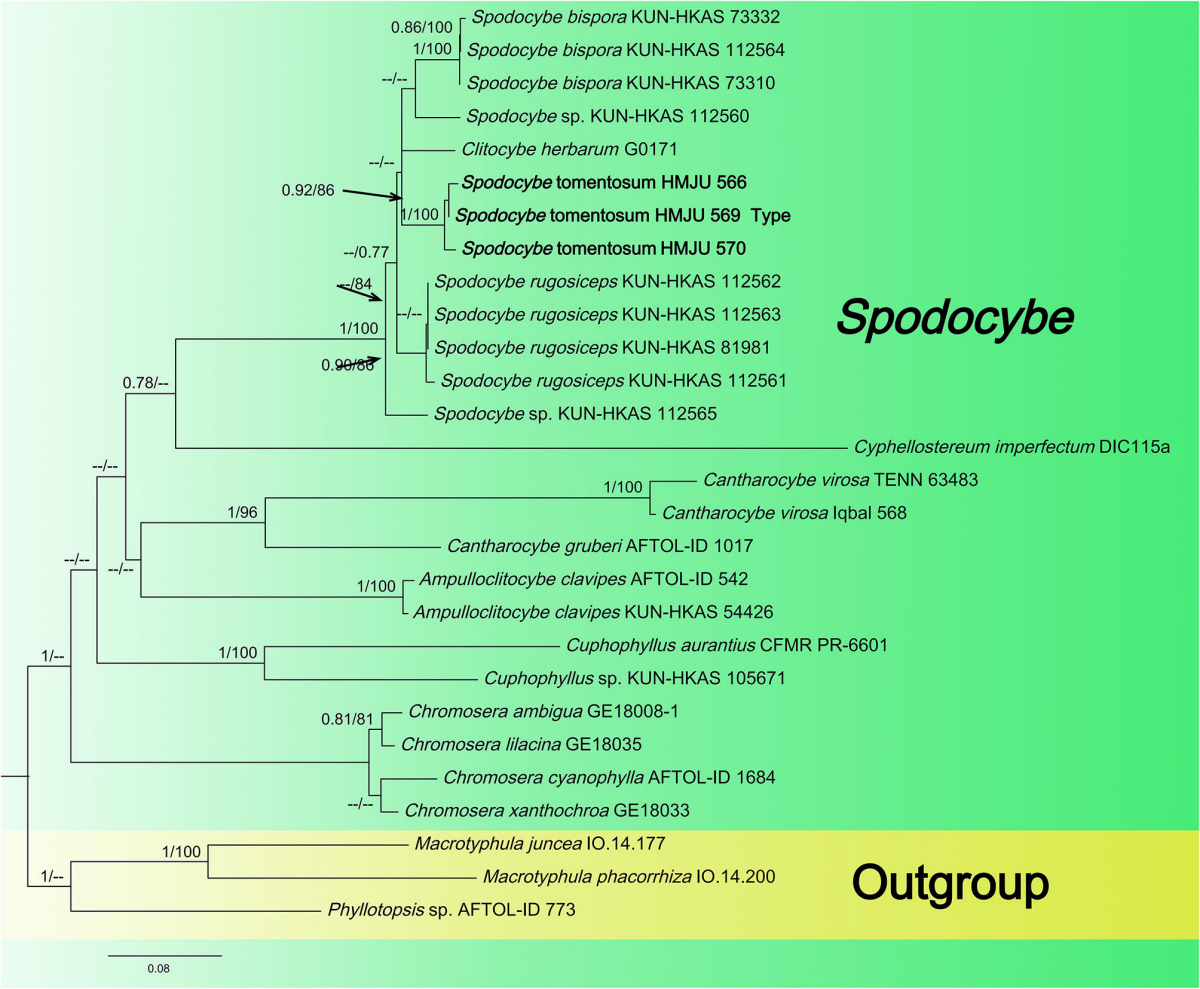
Maximum likelihood tree based on analyses of the ITS, nrLSU, *rpb2*, and *atp6* sequence data.

The newly obtained sequences were compared with representative ITS, *rpb2*, nrLSU, *atp6*, and *tef1-*α sequences retrieved from GenBank. According to the phylogenetic analysis, we selected *Pleurotus ostreatus* as an outgroup in Figure 1, *Mycena* aff. pura, *Panellus stipticus*, C*atathelasma imperiale, Tricholoma aurantium*, and *Tricholoma flavovirens* as outgroups in Figure 2, and *Macrotyphula juncea, Macrotyphula phacorrhiza*, and *Phyllotopsis* sp. as outgroups in Figure 3 (Raj et al., 2016; Daniëls et al., 2017). The sequences were aligned with MAFFT (Katoh and Standley, 2013) and then manually adjusted in Mega (Sudhir et al., 2016). The selection of the model was completed by ModelFinder based on the Bayesian information criterion (BIC) (Kalyaanamoorthy et al., 2017). Mapping and analyzing the phylogenetic position of the new species was done using maximum likelihood (ML) and Bayesian inference (BI) methods. The ML was calculated using IQ-TREE (Nguyen et al., 2015), and the BI phylogeny was inferenced by MrBayes 3.2.2 (Ronquist et al., 2012). The ML tree was evaluated by bootstrap analysis with 1,000 replicates (Stamatakis, 2006). A 50% majority-rule consensus cladogram was computed from the trees to obtain estimates for Bayesian posterior probabilities. The significance threshold was set to >0.95 for Bayesian posterior probability (PP) and >70% for ML bootstrap proportions (BP). All the sequences used in this study are listed in Table 2.

**Table 2:** List of fungal species and their information used in the phylogenetic analyses.

**Species**	**Collection**	**GenBank accession numbers**					**Location**
		**ITS**	**nrLSU**	***rpb2***	***tef1-**α*	***atp6***	
*Ampulloclitocybe clavipes*	AFTOL-ID 542	AY789080	AY639881	AY780937	–	–	–
	KUN-HKAS 54426	MW616462	MW600481	MW656471	–	–	China
*Cantharocybe gruberi*	AFTOL-ID 1017	DQ200927	DQ234540	DQ385879	–	–	USA
*C. virosa*	TENN 63483	KX452405	JX101471	–	–	–	India
	Iqbal 568	KX452403	KF303143	–	–	–	Bangladesh
*Chromosera ambigua*	GE18008-1	MK645573	MK645587	MK645593	–	–	France
*C. cyanophylla*	AFTOL-ID 1684	DQ486688	DQ457655	KF381509	–	–	USA
*C. lilacina*	GE18035	MK645577	MK645591	MK645597	–	–	Canada
*C. xanthochroa*	GE18033	MK645576	MK645590	MK645596	–	–	Canada
*Clitocella fallax*	O:O-F88953 52/85	KC816767	KC816936	–	KC816845	–	Norway
	CORT:25668OKM	KC816768	KC816937	–	KC816846	–	USA
	K:K(M) 116541 136 LP	KC816769	KC816938	–	KC816847	–	Spain
*C. mundula*	O:O-F71544 PM 67-95	KC816780	KC816950	–	KC816860	–	Norway
	CORT:7115 TJB	KC816781	KC816951	–	KC816861	–	USA
	CORT:7161 TJB	KC816782	KC816952	–	KC816862	–	USA
	O:O-F19454 20894	KC816784	KC816954	–	KC816864	–	Norway
*C. popinalis*	K:K(M) 146162 648/06	KC816795	KC816970	–	KC816877	–	UK
	O:O-F105360 116-2000	KC816800	KC816975	–	KC816881	–	Norway
	CORT:6378 TJB	KC816801	KC816976	–	KC816882	–	Switzerland
*C. herbarum*	G0171	–	MK277719	–	–	–	Hungary
*Cuphophyllus aurantius*	CFMR PR-6601	KF291099	KF291100	KF291102	–	–	Puerto Rico
*C*. sp.	KUN-HKAS 105671	MW762875	MW763000	MW789179	–	–	China
*Cyphellostereum imperfectum*	DIC115a	KF443218	KF443243	KF443277	–	–	Guatemala
*Hohenbuehelia angustata*	DAOM 195432a	MG383817	MG383827	–	–	–	Spain
	HMJAU4149	GQ142027	GQ142042	–	–	–	China
	CBS 856.85	MH861919	MG383826	–	–	–	Spain
	DAOM 195379	MG383815	MH873608	–	–	–	Netherlands
*H. atrocoerulea*	AMB 18080	KU355304	KU355389	–	–	–	Italy
	WU 8016	KU355309	KU355390	–	–	–	Italy
	AMB 18084	KU355301	KU355388	–	–	–	Italy
	AMB 18102	KY698000	KY698001	–	–	–	Italy
*H. auriscalpium*	WU 14662	KU355317	KU355391	–	–	–	Italy
*H. boullardii*	JCM06005	MG553637	MG553644	–	–	–	Spain
*H. carlothornii*	AMB 18106	KY698012	KY698013	–	–	–	Italy
*H. cyphelliformis*	Z+ZT 994	KU355325	KU355393	–	–	–	Italy
	AMB 18085	KU355324	KU355392	–	–	–	Italy
*H. faerberioides*	Mertens	MG553638	MG553645	–	–	–	Spain
*H. grisea*	MCVE 27293	KU355329	KU355394	–	–	–	Italy
	MCVE 23350	MH137807	MH137834	–	–	–	Spain
*H. ilerdensis*	Roux 3924	MG553639	MG553646	–	–	–	Spain
*H. josserandii*	P. K. EG10-812-T-F	KU355353	KU355403	–	–	–	Italy
	Roux 3270	KU355354	KU355404	–	–	–	Italy
*H. leightonii*	WU 5846	MG553640	MG553647	–	–	–	Spain
	WU 6004	MH137809	MH137835	–	–	–	Spain
	P. K. LP 311203-9	MH137810	MH137836	–	–	–	Spain
*H. longipes*	LIP 0400317	KU355333	KU355396	–	–	–	Italy
*H. mastrucata*	TRTC 152314	KU355336	KU355397	–	–	–	Italy
*H. petaloides*	TFB14021	KP026226	KP026217	–	–	–	USA
*H. reniformis*	HMJAU7091	GQ142024	GQ142041	–	–	–	China
*H. thornii*	AMB 18086	KU355342	KU355400	–	–	–	Italy
	WU 21790	KU355343	MG383831	–	–	–	Spain
	LIP 0401312	MG383823	NG060672	–	–	–	Europe
*H. tomentosa*	**HMJU00101**	**MK583558**	**MN947621**	–	–	–	**China**
	**HMJU00102**	**MK583564**	**MN382128**	–	–	–	**China**
*H. tremula*	WU 16294	KU355359	KU355407	–	–	–	Italy
	DAOM 180808	KU355357	KU355405	–	–	–	Italy
	M 0223665	KU355358	KU355406	–	–	–	Italy
*H. unguicularis*	Z+ZT 1112	KU355361	KU355408	–	–	–	Italy
	CBS 855.85	MH861918	MH873607	–	–	–	Netherlands
*H. valesiaca*	PDG 311005	KU355339	KU355398	–	–	–	Italy
	Roux 2975	KU355340	KU355399	–	–	–	Italy
	CL 990909/04	MH137819	MH137841	–	–	–	Spain
	JCS 600 B	MH137820	MH137842	–	–	–	Spain
*H. wilhelmii*	WU 7726	KU355300	KU355387	–	–	–	Italy
	BRNM 576483	MG383824	MG383832	–	–	–	Spain
	Z+ZT n. 1154	MF494947	MF494948	–	–	–	Italy
*Rhodocybe alutacea*	CORT:5726 TJB	KC816762	KC816931	–	KC816842	–	USA
*R. caelata*	CORT:6919 TJB	KC816764	KC816933	–	KC816843	–	USA
*R. collybioides*	CORT:10417 TJB	KC816766	KC816935	–	KC816844	–	Argentina
*R. fuliginea*	CORT:E537	KC816770	KC816940	–	KC816850	–	Australia
*R. hondensis*	CORT:6103 TJB	KC816771	KC816941	–	KC816851	–	USA
*R. lateritia*	CORT:E1589	KC816772	KC816942	–	KC816852	–	Australia
*R. mellea*	CORT:6883 TJB	KC816774	KC816944	–	KC816854	–	USA
*R. minutispora*	CORT:1071101-4	KC816777	KC816947	–	KC816857	–	Spain
*Rhodophana aershanensis*	**HMJU 760**	**OQ123823**	**OP919536**	**OP948886**		**OP948887**	
	**HMJU 812**	**OQ123821**	**OP919537**	**OP948888**		**OP948889**	
*R. guandishanense*	BJTC:FM0095	MT558552	MT558555	–	MT558558	–	China
	HSA 340	MT558553	MT558557	–	MT558560	–	China
	BJTC:FM0955	MT558554	MT558556	–	MT558559	–	China
*R. melleopallens*	O:O-F172919 415/83	KC816776	KC816946	–	KC816856	–	Norway
*R. nitellina*	O:O-F293352 HH74/10	KC816788	KC816958	–	KC816865	–	Norway
	O:Artsobs. 1541959	KC816790	KC816961	–	KC816868	–	Norway
	CORT:11CA025	KC816792	KC816965	–	KC816872	–	USA
*R. qinghaiensis*	**HMJU 380**	**OP919468**	**OP919476**	**OP948883**			
	**HMJU 5191**	**OQ123822**	**OP919551**	**OP948890**			
*R. stangliana*	CORT:2073TL	–	KC816992	–	KC816899	–	Denmark
*Spodocybe bispora*	KUN-HKAS 73310	MW762880	MW763005	MW789184	–	–	China
	KUN-HKAS 73332	MW762881	MW763006	MW789185	–	–	China
	KUN-HKAS 112564	MW762882	MW763007	MW789186	–	–	China
*S. rugosiceps*	KUN-HKAS 112561	MW762883	MW763008	MW789187	–	–	China
	KUN-HKAS 81981	MW762884	MW763009	MW789188	–	–	China
	KUN-HKAS 112562	MW762887	MW763012	MW789191	–	–	China
	KUN-HKAS 112563	MW762888	MW763013	MW789192	–	–	China
*S*. sp.	KUN-HKAS 112560	MW762889	MW763014	MW789193	–	–	China
	KUN-HKAS 112565	MW762890	MW763015	MW789194	–	–	China
*S. tomentosum*	**HMJU 566**	**OP935679**	**OP919483**	**OP948882**	–	**OP948891**	**China**
	**HMJU 569**	**OP935678**	**OP935676**	**OP948884**	–	**OP948892**	**China**
	**HMJU 570**	**OP935677**	**OP919526**	**OP948885**	–	**OP948893**	**China**
*Catathelasma imperiale*	CORT:11CA01A	KC816816	KC816994	–	KC816900	–	USA
*Macrotyphula juncea*	IO.14.177	MT232353	MT232306	MT242337	–	–	Sweden
*M. phacorrhiza*	IO.14.200	MT232363	MT232314	MT242347	–	–	France
*Mycena* aff. *pura*	CORT:11CA007	KC816817	KC816995	–	KC816901	–	USA
*Panellus stipticus*	CORT:11CA052	KC816818	KC816996	–	KC816902	–	USA
*Phyllotopsis* sp.	AFTOL-ID 773	DQ404382	AY684161	AY786061	–	–	–
*Pleurotus ostreatus*	AFTOL-ID 564	AY854077	AY645052	–	–	–	USA
	HKAS84903	KP867913	KP867901	–	–	–	Germany
	HKAS53480	KP867914	KP867902	–	–	–	Germany
*Tricholoma aurantium*	LCG2308	JN019434	JN019705	–	JN019386	–	Canada
*T. flavovirens*	CORT:11CA038	KC816819	KC816997	–	KC816903	–	USA

The GenBank accession numbers of ITS, nrLSU, *rpb2, tef1*-α, and *atp6* for our specimens were given in bold values.

## 3. Results

### 3.1. Phylogenetic analyses

A total of 282 sequences (107 ITS, 109 nrLSU, 29 *rpb2*, 33 *tef1-*α, 4 *atp6*) from 62 samples were used in the phylogenetic analyses, of which 31 (9 ITS, 9 nrLSU, 2 *tef1-*α, 7 *rpb2*, 4 *atp6*) were newly generated in the present study (Table 2). The newly generated sequences were submitted to GenBank (accession numbers are listed in Table 1). In this study, three datasets were analyzed: (i) namely the *Hohenbuehelia* dataset, (ii) the *Rhodophana* dataset, and (iii) the *Spodocybe* dataset.

#### 3.1.1. *Hohenbuehelia* dataset analyses

In this dataset, the final ITS dataset was composed of 46 sequences, which was 610 base pairs (bps) long and contained 540 (88%) conserved sites, whereas the nrLSU dataset was composed of 48 sequences, which was 798 base pairs long and contained 790 (98%) conserved sites. A total of 46 sequences were complete for both genes, and these datasets were combined for the phylogenetic analysis. The Bayesian PP value was 0.90 (left) and the MLBP value was 75% (right), and the nodes are annotated. The maximum likelihood tree is presented in Figure 1 with bootstrap values provided on the branches. Our sequences assembled together and fell under the genus *Hohenbuehelia*, which indicated that it should be a member of *Hohenbuehelia*. In particular, *H. tomentosa* formed a strongly supported branch in the phylogenetic trees where it occupies an independent but uncertain position with regard to the genus *Hohenbuehelia*.

#### 3.1.2. *Rhodophana* dataset analyses

Four *tef1-*α, four *rpb2*, and two *atp6* sequences of four specimens were newly generated in this study. The sequences were submitted to GenBank (accession numbers are listed in Table 1). The Bayesian and ML analyses resulted in a topology similar to the MP analysis, so only the ML tree was shown (Figure 2). Nodes were annotated if supported by >0.90 Bayesian PP (left) or >75% MLBP (right) values according to Vizzini et al. (2015) and Cai et al. (2020). For the ML analysis, sequences relating to 22 species were added. The ML tree represented in Figure 2 shows detailed results with high bootstrapping values. The outgroups used were *Mycena* aff. pura, *Panellus stipticus*, C*atathelasma imperiale, Tricholoma aurantium*, and *Tricholoma flavovirens*. The monophyly of the *Rhodophana* clade was strongly supported (BP = 100%, PP = 1.00), including *Rhodophana qinghaiensis* HMJU380 and *Rhodophana qinghaiensis* HMJU5191 (BP = 100%, PP = 1.00), *Rhodophana aershanensis* HMJU812 and *Rhodophana aershanensis* HMJU760 (BP = 100%, PP = 1.00), and four other *Rhodophana* species. *Rhodophana qinghaiensis* HMJU380 and *Rhodophana qinghaiensis* HMJU5191 independently separated a clade, and *Rhodophana aershanensis* HMJU812 and *Rhodophana aershanensis* HMJU760 also independently separated a clade (Figure 1). Four specimens fell under *Rhodophana*, which belonged to the genus *Rhodophana* and, therefore, should be a member of *Rhodophana*.

#### 3.1.3. *Spodocybe* dataset analyses

The *Spodocybe* dataset, consisting of 130 sequences dataset, was applied to phylogenetic analysis for displaying the relationships. *Macrotyphular juncea* (Alb. & Schwein.) Berthier., *Macrotyphula phacorrhiza* (Reichard) Olariaga et al., and *Phyllotopsis* sp. were used as the outgroups. Both BI and ML approaches resulted in the same tree topology; as such, only the ML tree was shown, with Bayesian PP values (left) and maximum likelihood tree BP values (right) provided near each node (Figure 3). In the three-gene tree (Figure 3), all new collections of *Spodocybe* from China fell into the *Spodocybe* clade and formed an independent clade. One collection (nrLSU: MK27771) from Hungary, labeled *Clitocybe herbarum* Romagn., fell under the *Spodocybe* clade, indicating that it should be a member of *Spodocybe*.

### 3.2. Taxonomy

#### 3.2.1. *Hohenbuehelia tomentosa* J. Z. Xu, sp. nov.

**Figure 4:**
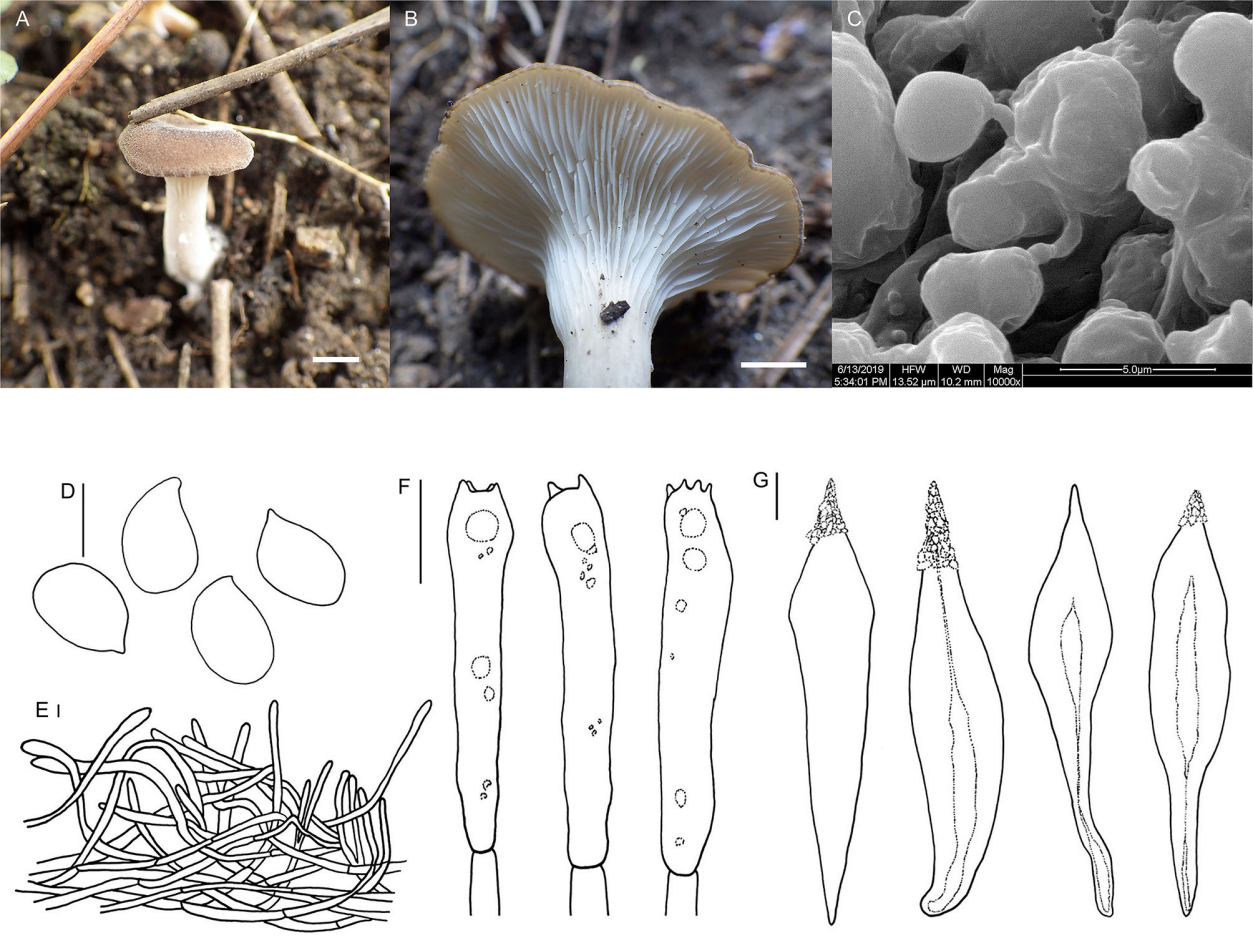
*Hohenbuehelia tomentosa* (HMJU00101, holotype). **(A, B)** Habitat and basidiocarps; **(C)** SEM images of basidiospores; **(D)** basidiospores; **(E)** pileipellis; **(F)** basidia; **(G)** Cheilometuloids. Bars: [(**A)** = 0.83 cm; **(B)** = 0.5 cm; **(D, F, G)** = 5 μm; **(E)** =2 μm].

Fungal Names number: FN 571264, Figure 4.

Diagnosis: Distinguished by a dark brown pileus with finely dense pure white tomentum; decurrent, pure white lamellae; a dirty white, subcentral stipe with a longitudinally fibrous striate surface; smooth, ellipsoid to oblong-ellipsoid spores; fusiform metuloid cystidia.

Holotype—China. Liaoning Province, Fumeng Town, Haitang Mountain, on humus or ground by the road, 6 August 2016, J. Z. Xu, HMJU00101.

Etymology—*tomentosa* (Lat.): referring to the pileus, which is always with finely dense pure white tomentum on the surface.

Description: Pileus 1.5–4.0 cm diam, subcircular, extremely depressed at the center, subfunnel-shape, margin incurved; surface with finely dense pure white tomentum at all ages, with radial stripes; gray-brown (7A3) to almost black, in some places dark brown (7E6) when young, dark brown (7E6) with a nearly black (6F6) center at a later stage; context thin; 1–2 mm thick at pileus. Lamellae 1.5–2.0 mm broad, decurrent, pure white, with fine pure white frosting at times, crowded, with one intercalated lamellula between each pair of lamellae reaching the stipe at times, edge concolorous, entire, even. Stipe 2–4 × 0.7– 0.9 cm, eccentric, broadened at base, and longitudinally fibrous striate; pale grayish beige (29A2) to dirty white (28A2). Smell and taste are insignificant.

Basidiospores (4.6) 5.0–7.1 (7.6) × (3.3) 3.6–4.4 (4.6) μm, Q = (1.44) 1.52–1.92 (1.98), (*n* = 30), ellipsoid to oblong-ellipsoid, hyaline, smooth, some with one or more oil drop, and inamyloid. Basidia (20) 22–25 (27) × 4.7–6.0 μm, clavate to subcylindrical, slightly broadened at apex, subhyaline, with two or four sterigmata, and sterigmata up to 2.1–3.6 (4.1) μm long. Thin-walled cystidia, rare. Metuloid cheilo- and pleurocystida abundant, cheilometuloids (41) 54–69 (76) × (8.8) 9.1–11.6 (14) μm, fusiform, subcylindrical to ventriculose, thick-walled, mostly with a narrow base and a lanceolate apical part, and incrusted with crystals at apex, some extremely broadened at the apex. Pleurometuloids similar to cheilometuloids. Hymenophoral trama is made up of regular, radially parallel hyphae, hyphae 2.5–5.6 μm wide, cylindrical, thin-walled, clamped, hyaline, smooth. Pileipellis is composed of thin-walled, branched, interwoven hyphae, hyphae 2.2–4.5 μm wide, tomentum, cylindrical, not or slightly constricted at the septa, hyaline, smooth.

Habitat: Scattered or single on humus or ground by the road. Known from Liaoning Province in China.

Additional specimen examined—China. Liaoning Province, Fumeng Town, Haitang Mountain, on soil under mixed forests dominated by Quercus mongolica 41°56′20.7^′′^N, 121°50′13.9^′′^E, 350 m, 6 August 2016, J. Z. Xu, HMJU00102.

Note: Morphologically, it is closely related to members of the subgenus *Hohenbuehelia* due to the eccentric stipe and smooth spores (Singer, 1986). *Hohenbuehelia longipes* (Boud.) M. M. Moser., *H. culmicola* Bon. and *H. ilerdensi*s Courtec., Vila & Rocabruna., share similar features with our species, whereas *H. culmicola* growing on culms and leaf-sheaths of *Ammophila arenaria* in coastal dunes produces a not translucent-striate, densely tomentose pileus, and a brown stipe covered in gray villose surface (Noordeloos, 1987). *H. longipes* mainly differs from *H. tomentosa* in having a longer stipe (40–60 (80) × 3–5 (8) mm in *H. longipes*, 20–40 × 7–9 mm in *H. tomentosa*), a brown-ochraceous to isabelline pileus and whitish-ochraceous gills (Thorn and Barron, 1986; Watling and Gregory, 1989). *Hohenbuehelia. ilerdensis*, growing at the bases of dead grasses in semi steppe communities, is distinguished from *H. tomentosa* in having a convex to flattened, smooth, violaceous to reddish brown pileus, a cylindrical, greyish stipe, and abundant cheilocystidia (Courtecuisse et al., 1999).

#### 3.2.2. *Rhodophana qinghaiensis* J. Z. Xu, sp. nov.

**Figure 5:**
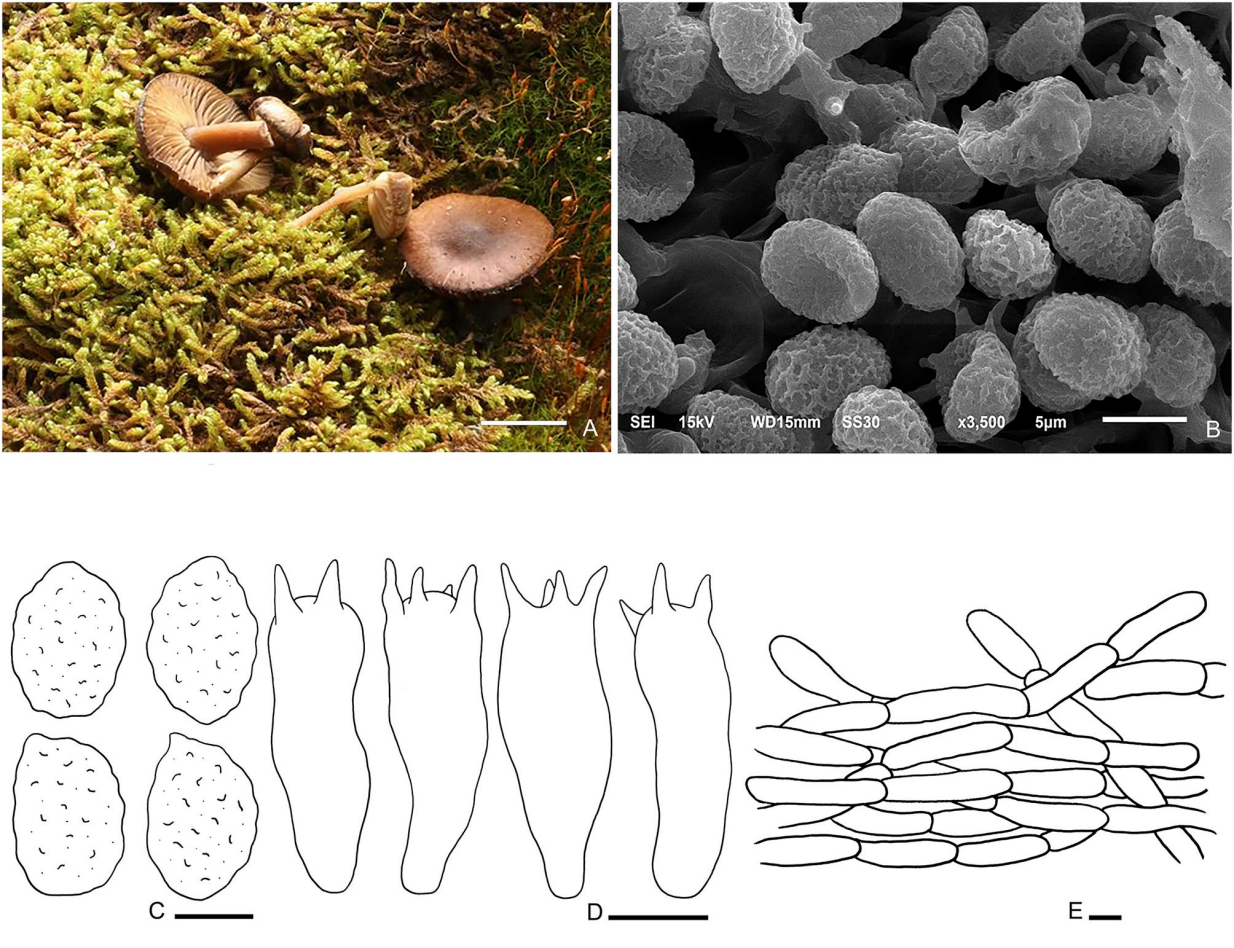
*Rhodophana qinghaiensis* (HMJU5191, holotype). **(A)** Habitat and basidiocarps; **(B)** SEM images of basidiospores; **(C)** basidia; **(D)** pileipellis; **(E)** basidiospores. Bars: [**(A)** = 1 cm; **(B, C)** = 5 μm; **(D, E)** = 10 μm].

Fungal Names number: FN 571265, Figure 5.

Diagnosis: Pileus reddish brown. Lamellar adnexed, gray-orange. Stipe light orange to reddish brown. Clamp connections are present. Basidiospores with strongly verrucose ornamentation all over.

Holotype—China. Qinghai Province, Haixi Korean, Halihatu National Forest Park, 37°2′0^′′^N, 98°39′32.4^′′^E, 3,604.1 m, 5 August 2018, J. Z. Xu, HMJU5191.

Etymology—*qinghaiensis* (Lat.): referring to the collection site.

Description: Pileus 1–2.5 cm in diam, applanate, umbo in the center, surface dry, not smooth, not hygrophanous; margin incurved becoming decurved, entire and regular; reddish brown (8E5) at the center, and brown-orange (7B5) at the margin. Lamellar adnexed, medium close, with lamellulae of 1–3 lengths; gray-orange (6B5). Edge concolor, entire, some broken in the middle, even. Stipe 2–3 × 0.3–0.6 cm, central, cylindrical, equal or slightly tapering toward the base, surface glabrous, smooth, solid; initially light orange (6B4), and becoming reddish brown (8D8) with age.

Basidiospores (5.9–) 6.2–8.2 (−8.6) × (4.1–) 4.5–5.9 (−6.5) μm, Q = 1.14–1.73, Qm = 1.4, broadly ellipsoid to ellipsoid in face view, oblong or amygdaliform in profile view, with strongly verrucose ornamentation all over, thin-walled, inamlyloid, brown in 3% of the KOH. Basidia (24.9–) 27.0–32.6 (−40.9) × 7.6–11.3 μm, clavate, mostly 4-spored, some 2-spored, sterigmata up to 4.9 μm long. Hymenophoral trama regular, hyphae cylindrical, hyaline, 6–16 μm wide. Pileipellis, a cutis of subparallel, cylindrical hyphae, hyaline, 3.2–9.0 μm in diam, thin-walled. Clamp connections are present.

Habitat: Scattered on the moss in coniferous forest.

Additional specimen examined—China. Qinghai Province, Haixi Korean, Halihatu National Forest Park, 5 August 2018, J. Z. Xu, HMJU380.

Notes: The key characteristics of *R. qinghaiensis* are reddish brown pileus, margin incurved becoming decurved, entire, gray-orange and adnexed lamellar, entire or broken in the middle, light orange to reddish brown, and smooth and solid stipe. *Rhodophana flavipes* T. J. Baroni, P. P. Daniëls & O. Hama. differs from *R. qinghaiensis* in the pileus with fibrils and squamules, adnexed lamellar and light yellow, and hollow stipe with reddish bruising of fibrils; *Rhodophana squamulosa* K. P. D. Latha & Manim. also have gray-orange lamellar, but *R. squamulosa* have squamules on the pileus. In addition, *R. squamulosa* has adnexed lamellar and hollow stipe with appressed fibrillose all over.

#### 3.2.3. *Rhodophana aershanensis* J. Z. Xu, sp. nov.

**Figure 6:**
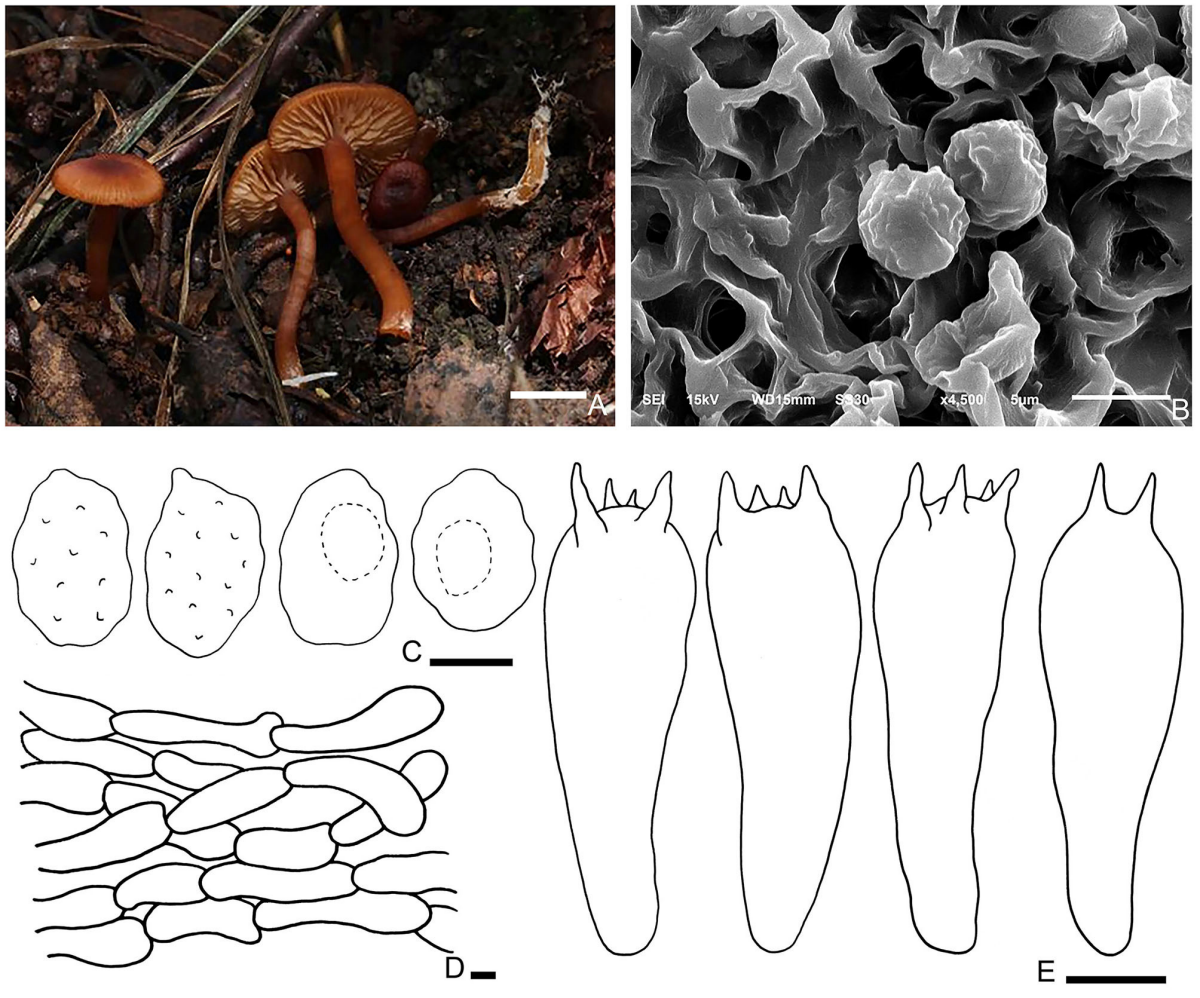
*Rhodophana aershanensis* (HMJU760, holotype). **(A)** Habitat and basidiocarps; **(B)** SEM images of basidiospores; **(C)** basidiospores; **(D)** pileipellis; **(E)** basidia. Bars: [**(A)** = 1 cm; **(B, E)** = 5 μm; **(C)** = 3 μm; **(D)** = 10 μm].

Fungal Names number: FN 571266, Figure 6.

Diagnosis: Pileus dark brown or reddish brown, with striate incurved margin. Lamellar light orange, decurved. Stipe solid, with white basal mycelial cord. Clamp connections, present. Basidiospores undulate-pustulate, inamyloid.

Holotype—China. Neimenggu Autonomous Region, Aershan City, Bailang Town, 18 August 2020, J. Z. Xu, HMJU760.

Etymology—*aershanensis* (Lat.): referring to the collection site.

Description: Pileus 1–2 cm in diam, smooth, slightly hygrophanous when young, gradually becoming dry with age; umbo in the center, depressed at sides when young, applanate with a small, obtuse bulge when mature, the depression becoming subsulcate with expansion; margin striped, incurved, regular, even; initially dark brown (8F6) at the center, margin reddish brown (9E8), reddish brown (9E7) with age, brown-orange (7B7) toward the margin. Lamellar decurrent, medium crowded, with lamellulae of 3–4 lengths; edges concolor, entire and even; light orange (7C6). Stipe 2–3.5 × 0.3– 0.5 cm, central, cylindrical, curved toward apex, equal or slightly tapering toward the base, glabrous, smooth, solid, with white basal mycelial cord; dark brown (9F6) at first, reddish brown (8D8) with age, and light orange (7C7) at the base.

Basidiospores (1.3–) 2.7–5.8 (−6.6) × (1.2–) 2.2–5.1 (−6.2) μm, Q = 1.04–1.39, Qm = 1.1, lacrymoid (ellipsoid with suprahilar depression), the surface undulate-pustulate, slightly undulate-pustulate all over, thin-walled, hyaline, with a large guttula, inamyloid. Basidia 20.8–24.6 (−26.4) × (6.7–) 7.2–8.9 (−9.6) μm, clavate, hyaline, mostly tetrasporic, sterigmata up to 2.5 μm long. Hymenophoral trama regular, hyaline, cylindrical hyphae, 2–9 μm wide. Pileipellis, a cutis of subparallel, dense, cylindrical hyphae, hyaline, 3.2–14.6 μm in diam., and thin-walled. Clamp connections are present.

Habitat: Scattered on soil in mixed forests.

Additional specimen examined—China. Neimenggu Autonomous Region, Aershan City, Bailang Town, 18 August 2020, J. Z. Xu, HMJU812.

Notes: *Rhodophana flavipes* resembles *Rhodophana aershanensis* due to the reddish-brown pileus, and light orange lamellar, but *R. flavipes* has scaly, hygrophanous pileus, adnexed lamellar, and light yellow, hollow stipe; *Rhodophana canariensis* (Dähncke, Contu, and Vizzini) T. J. Baroni & Bergemann. showed similarities with *Rhodophana aershanensis* in the appearance of striations on the pileus, however, *R. canariensis* is differentiated by tan and hygrophanous pileus, light pink and adnate lamellar, and stipe with orange basal tomentum.

#### 3.2.4. *Spodocybe tomentosum* J. Z. Xu, sp. nov.

**Figure 7:**
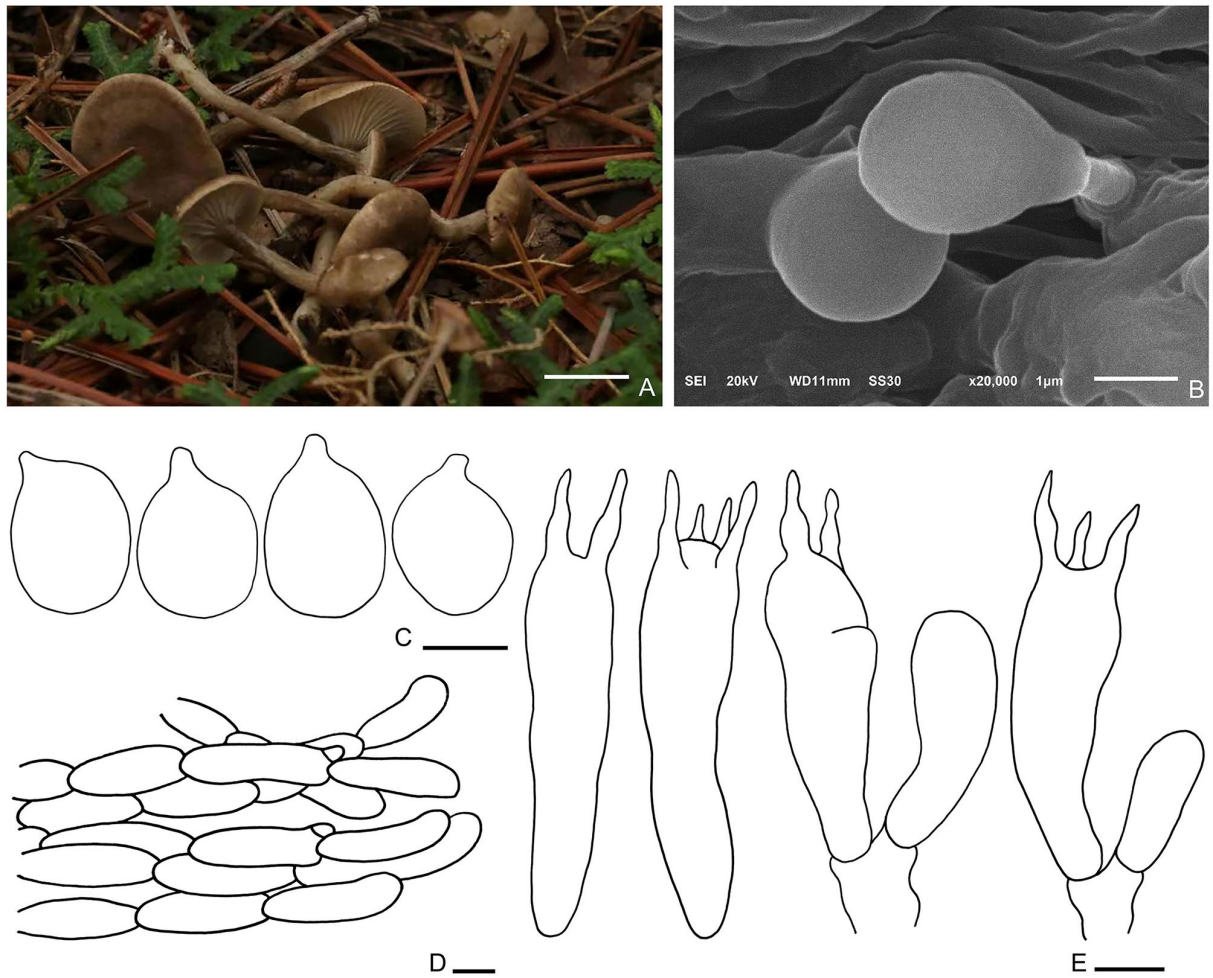
*Spodocybe tomentosum* (HMJU569, holotype). **(A)** Habitat and basidiocarps; **(B)** SEM images of basidiospores; **(C)** basidiospores; **(D)** pileipellis; **(E)** basidia. Bars: [**(A)** = 1 cm; **(B)** = 1 μm; **(C)** = 3 μm; **(D)** = 10 μm; **(E)** = 5 μm].

Fungal Names number: FN 571267, Figure 7.

Diagnosis: Basidiomes small, subclitocyboid. Pileus 1.5–3 cm in diam, at first applanate, then concave. Lamellae decurrent. Stipe central, hollow. Basidiospores are broadly ellipsoid to ellipsoid, hyaline, smooth, thin-walled, and inamyloid. Clamp connections were abundant.

Holotype—China. Liaoning Province, Fuxin City, Haitang Mountain, 41°53′50.6^′′^N, 121°47′18.3^′′^E, 428.8 m, 9 August 2019, J. Z. Xu, HMJU569 (holotype).

Etymology—*tomentosum* (Lat.): referring to the pileus, which was always with finely dense pure white tomentum on the surface.

Description: Basidiomes small, subclitocyboid. Pileus 1.5–3 cm in diam, at first applanate, then concave; with finely dense pure white tomentum on the surface; brown (7F7) to dark brown (7E6), gradually fading toward the margin; with papillae to applanation in the center. Lamellae decurrent, relatively crowded, with intercalated lamellulae, sometimes forked, concolorous with margin. Stipe 1.5–3.5 × 0.3–0.5 cm, equally thick, central, hollow; surface nearly smooth or slightly longitudinal stripe, concolorous with pileus.

Basidiospores (3.1–) 3.3–4.9 (−5.4) × 2.2–3.8 μm, Q = 1.22–1.55, Qm = 1.4, broadly ellipsoid to ellipsoid, hyaline, smooth, thin-walled, inamyloid. Basidia (19.9–) 23.6–27.6 × 5.4–6.7 μm, clavate, hyaline; 4-spored majority, 2-spored minority, sterigmata up to 4.4 μm long. Cystidia absent. Hymenophoral trama regular, hyphae cylindrical, hyaline, 1–7 μm wide. Pileipellis, a cutis, cylindrical, hyphae 9–10 μm wide, enlarged at the end. Clamp connections are abundant.

Habitat: Scattered on soil in coniferous forests.

Additional specimen examined—China. Liaoning Province, Fuxin City, Haitang Mountain, 9 August 2019, J. Z. Xu, HMJU566; Liaoning Province, Fuxin City, Haitang Mountain, 9 August 2019, J. Z. Xu, HMJU570.

Note: *Spodocybe tomentosum* differs from *S. rugosiceps* in that the pileus never forms rugosity during growth. Microscopically, *S. tomentosum* is mainly different from *S. rugosiceps* in having broadly ellipsoid to ellipsoid basidiospores. *Spodocybe tomentosum* and *S. bispora* can be differentiated by the broadly ellipsoid to ellipsoid basidiospores with Q = 1.22–1.55 (basidiospores cylindrical Q = (2.05) 2.11–3 (3.33) in *S. bispora*) and 2 to 4 spored basidia (He and Yang, 2021).

## 4. Discussion

In the Index Fungorum, *Hohenbuehelia* contains 135 valid records, *Rhodophana* contains 13 valid records, and *Spodocybe* contains 6 valid records. In addition, *R. guandishanense, S. bispora*, and *S. rugosiceps* were published in China. Our present study demonstrates a new species of *Hohenbuehelia*, two new species of *Rhodophana*, and a new species of *Spodocybe* based on both morphological characteristics and molecular phylogenetic analyses.

In the ITS + nrLSU tree, *H. tomentosa* formed an independent lineage and grouped with *H. auriscalpium* (Maire) Singer. *Hohenbuehelia auriscalpium* lacks a subcentral, fibrous striate stipe, and the cheilometuloids incrusted with crystals in *H. auriscalpium* are rare (Singer and Kuthán, 1980). In addition, *H. longipes, H. tremula* (Schaeff.) Thorn & G. L. Barron., *H. wilhelmii* Consiglio & Setti., *H. angustata* (Berk.) Singer., *H. thornii* Consiglio & Setti., and *H. petaloides* (Bull.) Schulzer are closely related to *H. tomentosa*. Morphologically, *H. longipes* mainly differs from *H. tomentosa* in having a longer stipe (40–60 (80) × 3–5 (8) mm in *H. longipes*, 20–40 × 7–9 mm in *H. tomentosa*), a brown-ochraceous to isabelline pileus, and whitish-ochraceous gills (Thorn and Barron, 1986; Watling and Gregory, 1989). *Hohenbuehelia petaloides, H. angustata, H. wilhelmii*, and *H. tremula* all lack a subcentral, fibrous striate stipe (Singer and Kuthán, 1980; Thorn and Barron, 1986; Corner, 1994; Consiglio et al., 2018). *Hohenbuehelia thornii* has a gelatine layer in its pileipellis, which is absent in *H. tomentosa* (Consiglio, 2017).

The key characteristics of *R. qinghaiensis* are reddish brown pileus, margin incurved becoming decurved, entire, gray-orange and adnexed lamellar, and entire or broken in the middle, light orange to reddish brown, smooth and solid stipe. The main characteristics of *R. aershanense* are the dark brown or reddish-brown pileus with striate incurved margin, light orange and decurved lamellar, and dark brown or reddish-brown and solid stipe with white basal mycelial cord. In the phylogenetic tree, *R. qinghaiensis* forms an independent lineage and is grouped with *R. stangliana* (Bresinsky and Pfaff) Vizzini. *Rhodophana aershanensis* formed an independent lineage. *Rhodophana qinghaiensis* differs from *R. aershanensis* by having the pileus without hygrophanous, lamellar adnexed, basidiospores without guttula, and sterigmata longer. *Rhodophana guandishanensis* differs from *R. qinghaiensis* in that it has orange-yellow and not-striate pileus, decurrent lamellar, and hollow stipe. *Rhodophana guandishanensis* resembles *R. aershanensis* due to its decurrent lamellar, but *R. guandishanensis* has an orange-yellow and not-striate pileus, light yellow lamellar, and light orange and hollow stipe.

*Spodocybe tomentosum* is characterized as subclitocyboid and small basidiomes, decurrent lamellae, central stipe, broadly ellipsoid to ellipsoid and inamyloid basidiospores, and abundant clamp connections. In the ML tree, *S. tomentosum* formed an independent lineage. In addition, *Cantharocybe* and *Ampulloclitocybe* also have a good genetic relationship. *Spodocybe tomentosum* differs from *S. rugosiceps*, in that the pileus never forms rugosity during growth. *Spodocybe tomentosum* is mainly different from *S. rugosiceps* in having broadly ellipsoid to ellipsoid basidiospores. *Spodocybe tomentosum* and *S. bispora* can be differentiated by the broadly ellipsoid to ellipsoid basidiospores with Q = 1.22–1.55 (basidiospores cylindrical Q = (2.05) 2.11–3 (3.33) in *S. bispora*) and 2 to 4 spored basidia (He and Yang, 2021).

In summary, our study used morphological characteristics and phylogenetic analysis to identify four new species, namely *H. tomentosa, R. qinghaiensis, R. aershanensis*, and *S. tomentosum*.

## Data availability statement

The datasets presented in this study can be found in online repositories. The names of the repository/repositories and accession number(s) can be found in the article/supplementary material.

## Author contributions

JX and TW wrote the manuscript. JX, YJ, XL, and DZ carried out experiments. MIH revise manuscript. JX collecting specimens and designed experiments. All authors contributed to the article and approved the submitted version.
